# Effects of the NeuroHAB Program on Low Back Pain and Oswestry Disability Index Scores: A Retrospective Wait-List Control Study

**DOI:** 10.3390/jfmk9030118

**Published:** 2024-07-03

**Authors:** Brogan Williams, David Johnson

**Affiliations:** The Back Pain and Functional Movement Training Centre, Brisbane, QLD 4102, Australia

**Keywords:** low back pain, ODI, NeuroHAB, functional movement therapy, exercise, back pain rehabilitation

## Abstract

Movement theory and the study of movement dysfunction mark a paradigm shift in the treatment of low back pain symptoms, the majority of which are mechanical in origin at the outset. Treating movement dysfunction centers around unified and consistent rehabilitation that defines proficient movement for the lumbopelvic spine. The purpose of this study is to document the improvement in pain and disability of 290 patients who underwent NeuroHAB Functional Movement Therapy to reverse their lumbopelvic movement dysfunction attributed to causing their back pain symptoms between 2019 and 2023. Oswestry Disability Index (ODI) scores were collected from each participant on three occasions: the first consultation, after a waiting period/pre-intervention, and after the eight-week intervention. A single-factor ANOVA of all three ODI data sets was conducted, along with supporting descriptive statistics. A post-hoc *t*-test pairwise comparison was conducted for accuracy. The average ODI 1 score (taken at the first consultation) was 15.26 ± 6.1% (CI: 14.3–16.2); ODI 2 (after a waiting period, before NeuroHAB) was 14.71 ± 6.0% (CI: 13.82–15.59); and ODI 3 (post-intervention) was 9.09 ± 8.6% (CI: 8.305–9.875). There was no significant change from ODI 1 to ODI 2 (between the consultation and waitlist control periods). However, a significant reduction between ODI 2 and ODI 3 was observed (pre- and post-intervention) (mean difference: 5.62, *p* ≤ 0.001), and a 40.41% reduction was observed between ODI 1 (the ODI score taken at the first consultation) and ODI 3 (the ODI score taken after NeuroHAB, post-intervention) (mean difference: 6.17, *p* ≤ 0.001). A 50% ODI reduction was reported in the “Crippled” category (mean difference 16.15, *p* ≤ 0.001). The inclusion of functional movement proficiency and stability in future guidelines is a necessary step towards meaningful improvement in epidemic levels of back pain-related clinical and economic morbidity.

## 1. Introduction

As a leading burden of disease globally, the devastating clinical and economic effects of chronic low back pain (LBP) are undeniable and non-conjecturally asserted in every review of the topic [1]. LBP is not only the most prevalent musculoskeletal health complaint seen by general practitioners but also one of the leading causes of musculoskeletal disability worldwide [2,3,4]. In 2020, 619 million people were affected by LBP, with a projected increase to 843 million by 2050 [5,6].

According to Bardin et al. (2017), the current goal of LBP diagnostic triaging is to rule out immediate red flags or spinal pathology (this constitutes < 1% of cases), followed by radicular syndrome (5–10% of cases) and the rest, fumbled into the medical abyss of non-specific low back pain (NSLBP), which represents over 90% of cases [2]. Worldwide guidelines focus on patient education/reassurance, passive modalities, returning to regular activity and physical exercise, and using non-steroidal anti-inflammatory drugs (NSAIDs) and opioid analgesics cautiously [7,8]. However, these guidelines clearly fall short. With the clinician or therapist using a classification diagnostic term so broad as “NSLBP”, the treatment often becomes very “non-specific” and often ineffective. Pain is a symptom, not a diagnosis, and should not be used interchangeably as a disease entity, as the term non-specific lower back pain implies. This compromises the global therapeutic community’s understanding of causation, resulting in LBP becoming a condition that is benign on the surface yet ubiquitous and disabling due to its ability to evade definitive diagnosis. In the absence of a disease, a diagnosis, and a cause, the likelihood of implementing an effective therapy or cure is low, as supported by the WHO and 2018 Lancet LBP review data [1,3].

### 1.1. Movement Dysfunction and Movement Theory

**Figure 1:**
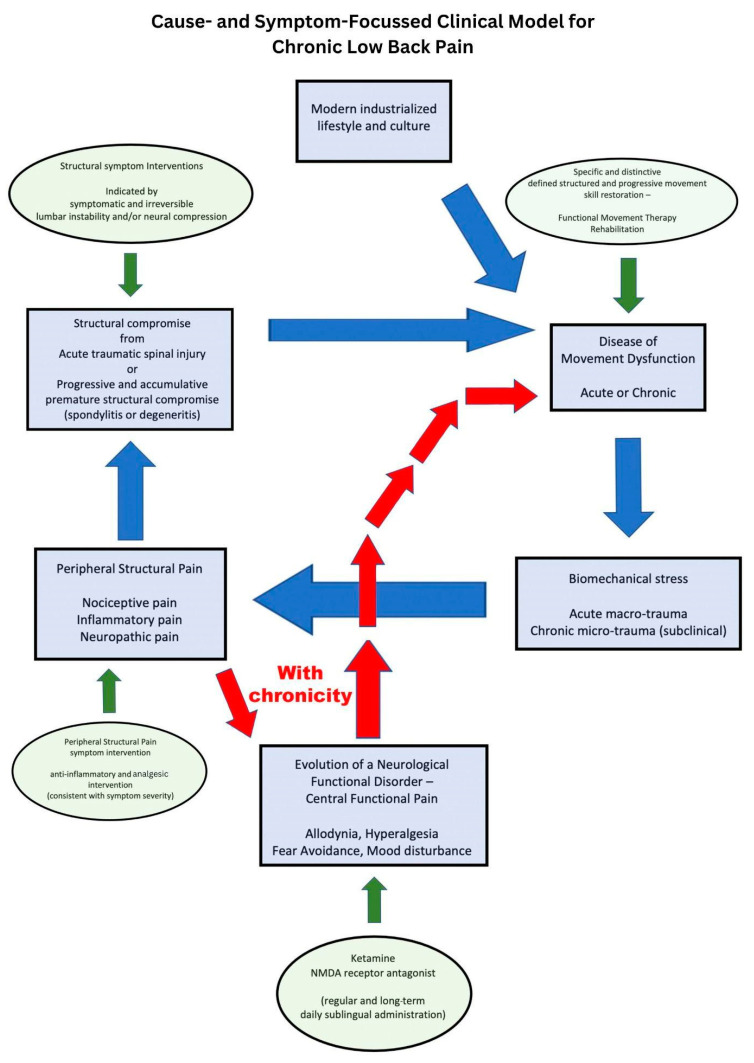
Cause- and symptom-focused clinical model for chronic low back pain.

Movement theory and the study of movement dysfunction open up a paradigm shift in treatment revolving around unified and consistent rehabilitation that defines proficient movement for the lumbopelvic spine. Lumbopelvic bending movement is proficient from childhood but becomes corrupted through the consistent pressure of biomechanical insults from a modern lifestyle. The essential functional musculoskeletal requirements to push, pull, climb, squat, hinge, press, and lift are all but eliminated in today’s industrialized modern society. Ever-shrinking non-industrialized cultures have far less chronic musculoskeletal disease, particularly low back pain symptoms, despite the dramatic differences in health care and rehabilitation services [9,10,11]. Consider the accumulative effect on the musculoskeletal system of eight hours a day of office work or scholastic education, the convenience of motorized transport, and a modern kitchen for food preparation. The active, passive, and subsequent neural control subsystems that determine spinal joint stability are compromised in modern humans by this metaphorical “domestic cage” of the modernized world [10,11]. Discretionary functional capacity, domestic athleticism, or sporting athleticism can be elevated on a foundation of mandatory joint stability obtained by movement proficiency, which reflects the neural control subsystem described by Panjabi [12,13] (see Figure 1).

### 1.2. Functional Movement and Functional Capacity

Functional movement, as defined in this study, can be characterized as universally natural, core-to-extremity, compound multi-joint movement, which is inherently the most biomechanically efficient means of performing mathematically calculable work (work = load × distance) [14]. This implies that when the human musculoskeletal system expresses functional movement, it is most capable of moving the heaviest loads at the largest distance quickly, repetitively, and for the longest duration [15]. By expressing functional movement as a default for activities of daily living (be they trivial or physically demanding), these activities promote the ongoing enhancement of musculoskeletal conditioning and functional capacity [15,16,17,18,19]. One’s capacity for competent and functional movement is considered by many a fundamental metric of health [18,19]. It determines the functional demands an individual can meet, which not only reflect their quality of life but also influence joint stability by virtue of the direct relationship between an individual’s functional capacity and joint stability and, conversely, the inverse relationship between functional demands and stability [15,16,17,18,19,20].

### 1.3. Movement Proficiency

NeuroHAB movement points of performance in relation to the lumbopelvic spine define how to express functional movement with proficiency. Five key points of performance are used to govern how the lumbopelvic region should function; these include 1. hip-centric rotation, 2. the maintenance of a neutral lumbar spine, 3. posterior kinetic chain-driven movement, 4. knee positions that are unloaded, and 5. proficiency in limited intensity of movement. Default movement proficiency points of performance for activities of daily living need to be clearly defined and then transferred to the back pain patient through skilled movement therapy [21]. Lifestyle functional demands become restricted to keep capacity and demand in check. In clinical practice, this is observed as a decreased quality of life, or if functional demands cannot be restricted (for example, employment tasks), potent symptom-based analgesics steadily escalate. Patients are provided with the opportunity to restore these fundamental functional movement skills, apply them to their infinite daily life activities, and acquire the ability to rehabilitate their back pain symptoms through their daily movement activities, enabling them to progressively build functional capacity, prevent relapsing pain, and improve quality of life by eliminating the disease of movement dysfunction.

One may look to commonly performed motor control and exercise physiology, posture training, and physio-Pilates for guidance in this field; however, all of these movement and stability-based approaches have been disappointing in managing chronic low back pain [10,22,23,24]. Upon deeper interrogation of these methods, they cannot be regarded as meeting the strict criteria for effective central nervous system motor pattern rehabilitation. They are overtly non-functional in their implementation. General exercise does not necessarily translate to more proficient and skilled functional movement, hence the observed disappointing outcomes obtained for treating back pain symptoms with these methodologies and motor control exercise in particular [4,5,9,10,23,24,25,26].

### 1.4. NeuroHAB

Delivered within an 8-week movement therapy program called NeuroHAB, proficient spinal biomechanics and movement patterns are used to restore central nervous system-derived motor patterns, reversing movement dysfunction of bending and establishing new default motor skills for functional movement tasks. On the foundation of movement proficiency and less biomechanically derived pain, functional capacity can be progressively restored to meet the requirements for individual lifestyle functional demands and the basic activities of daily living [27]. NeuroHAB is a term used to distinguish itself as a central nervous system motor pattern-focused, functional, and qualitative coordination of movement approach rather than a more conventional, structural, and quantitative musculoskeletal-focused approach.

It has also been stated that treatment interventions must address the cause of the pain, which is primarily functional. The majority of industry-wide rehabilitation services focus on addressing structure-related symptoms and ignore function-related causes. Until we start to change our mindset and our framework for understanding the condition of back pain, the statistics and the data will continue to worsen over time rather than improve, despite widely available rehabilitation services for back pain symptoms [28]. 

### 1.5. Lumbopelvic Proficient Movement

Movement proficiency for the lumbopelvic spine has not been clearly defined in published, peer-reviewed literature. We define movement proficiency criteria as follows [27].

#### 1.5.1. Hip-Centric Rotation, Minimizing Intra-Lumbar Centric Flexion for Forward Bending Tasks

The hip is a ball-and-socket joint with an anatomical form that functions optimally for rotation. The intersegmental lumbar spinal tripod joint system of the two zygapophyseal joints and the disco-vertebral joint is susceptible to accelerated structural deterioration with repetitive flexion and extension [21,23,26,27].

#### 1.5.2. Neutral Spine Maintenance during Forward Bending Tasks

The multifidus muscle is the most important intersegmental stabilizer of the lumbar spine. Its concentric contraction function extends the lumbar spine into neutral lordosis and prevents anterior subluxation. This critical stability-focused muscle group is conditioned predominantly through hip-centric hinging with a neutral lumbar lordotic-positioned spine [29,30,31,32,33,34]. This is commonly called a hip hinge when a shallow range of motion is the movement task. If a greater range of motion is required, a proficient squat or lunge is performed to maintain this proficient movement criterion.

#### 1.5.3. Posterior Kinetic Chain-Powered Movement

The majority of the lumbar spine is situated behind our center of gravity, and modern humans have adopted a bipedal upright gait for evolutionary advantages [35]. However, this has increased our susceptibility to movement dysfunction and deconditioning of the posterior kinetic chain in contrast to our quadrupedal ancestors with highly developed posterior chain musculature, particularly the gluteal and hamstring muscles [35]. Fundamentally, the repetitive activity of the musculoligamentous and elastic posterior kinetic chain, which includes stabilizer and mobilizer muscles acting on the lumbar spine, enhances its condition, affording optimal stability and functional capacity to the lumbopelvic spine.

#### 1.5.4. Unloaded Knee Position

During bending tasks, a conscious effort to avoid loading the knees through anterior kneeling kinematics ensures persistent activation of the posterior kinetic chain [36]. Comparatively, a kneel versus a squat displays measurable differences in knee joint loading [37]. The extreme and end range of kneeling is balancing on tip-toes with heels raised. This effectively breaks the musculoligamentous tension band and power of the posterior kinetic chain, leading to accumulative deconditioning if this becomes a default movement pattern. In contrast, enhancement of the posterior kinetic chain is asserted by the adoption of unloaded knee positions, with the hips translating posteriorly and thereby activating the posterior kinetic chain during bending tasks [36,37].

#### 1.5.5. Proficiency-Limited Execution of Movement Intensity

If proficiency through movement points of performance 1–4 becomes corrupted due to the demand of the movement task, be it due to factors influencing intensity such as range of motion, repetitions, load, duration, or speed, the task intensity needs to be scaled back or regressed to maintain proficiency. Over time, with consistent default expression of movement proficiency and the resolution of movement dysfunction, we assert that a steady improvement in the health and condition of the musculoskeletal system is expected. With the improvement in condition and health of the musculoskeletal system pertaining to the lumbopelvic spine, we assert that the range of motion, load, speed, and duration of movement tasks will become more favorable. Fundamentally, this equates to greater functional capacity and quality of life.

These criteria for proficient movement were chosen because they represent the performance points for default biomechanically stable and, therefore, healthy lumbopelvic bending tasks. We propose that for a musculoskeletal pain symptom to reach epidemic proportions, as is the case with low back pain, a patho-anatomical and ubiquitous cause must exist. Without this, the condition would never reach epidemic status. The disease of movement dysfunction, as described by Johnson and Kim (2023), meets that criterion as a prevalent, ubiquitous disease process afflicting ninety percent of individuals working and living in modern society in a subclinical manner [38].

Repetitive biomechanical stress incites pain and disability through mechanisms producing biological inflammation and activated nociception. Painful movement fundamentally and logically potentiates further dysfunctional movement, reinforcing the “pain–dysfunction” cycle.

The aim of this study is to document the improvement in pain and disability of 290 patients who underwent NeuroHAB Functional Movement Therapy to reverse their lumbopelvic movement dysfunction attributed to causing their back pain symptoms between 2019 and 2023.

## 2. Methods

### 2.1. The NeuroHAB Program

The NeuroHAB intervention is eight weeks long and comprises two one-hour weekly sessions. Every session is movement-focused with no manual, passive, or recumbent therapy. The classes are structured in a progressive and reproducible format with the ability to be administered in multiple locations by adequately skilled movement therapists familiar with the methodology and teaching techniques that involve a combination of verbal, visual, and tactile cues designed to assist the patient’s acquisition of NeuroHAB-defined movement. The objectives of the 8-week, 16 h program (2 × 1 h movement therapy sessions per week) are to restore default upright bending biomechanics that express NeuroHAB movement points of performance (as defined above). One or two movement therapists instruct the movement performance of the class (ranging from 1–12 patients) with visual, verbal, and tactile cues. Regular and frequent correction is required in the pursuit of the virtuosity of simple functional bending movements. Movements progress over the duration of the program, commencing with the hip hinge (assisted with the application of a dowel and 3 points of contact), an awareness of posterior kinetic chain activation, and then simple walking with gluteal muscle activation.

**Figure 2:**
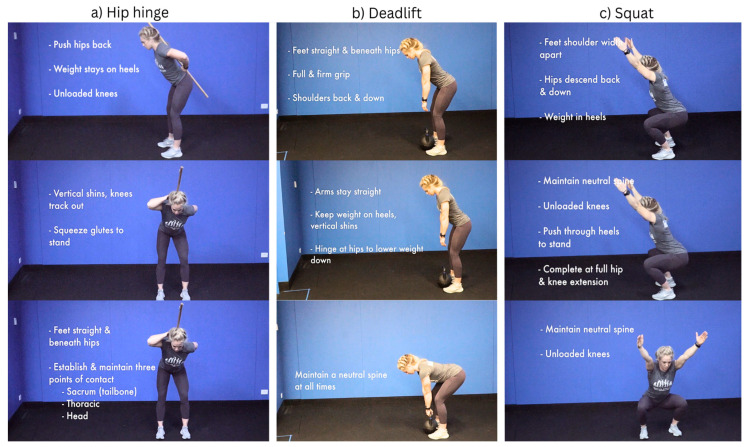
NeuroHab movement examples.

Over the eight weeks of movement therapy, coordination increases steadily with less requirement for correction and improved cadence or speed of tasks, range of motion, duration, and power. Patients receive specific guidance and participate actively and repetitively in movement drills that promote the development of default motor patterns that express movement proficiency for daily living and bending activities, characterized by hip-centric rotation with neutral spine awareness in the presence of an active posterior kinetic chain. These movement characteristics eliminate knee loading and anterior chain-dominant movement, such as kneeling, which inherently deactivates the posterior kinetic chain, promoting a loss of undesirable stable and neutral spinal positions (see Figure 2) [15,28,38].

### 2.2. Study Design

**Figure 3:**
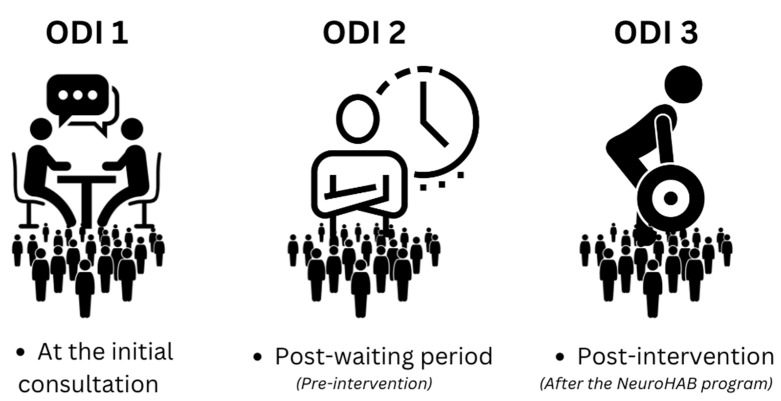
Each phase is represented by an ODI score.

A single neurosurgeon referred all patients in the study to one of two rehabilitation centers offering NeuroHAB for chronic low back pain. This has been the usual standard of care for the referring clinician since 2015. Over the four-year period between 2019 and 2023, 290 patients participated in NeuroHAB Functional Movement Therapy and completed the necessary pre- and post-NeuroHAB intervention ODI assessments. ODI scores were collected on three occasions across the span of this study: one at the first consultation with the referring neurosurgeon, a second at the commencement of the NeuroHAB Functional Movement Therapy intervention, and a third and final after the eight-week intervention. The time period on the waiting list to commence the NeuroHAB intervention varied between 3 weeks and 3 months. From this data, a “waiting list” control–comparison group change in average ODI score was calculated. Absolute improvements in average ODI scores were observable by the comparison of post-intervention ODI 3 with pre-intervention ODI 1 and ODI 2. Patients remained under their own control while receiving usual care on the “waiting list” before the NeuroHAB Functional Movement Therapy intervention. ODI 1 is representative of the ODI score each patient received upon the first consultation. ODI 2 is representative of the ODI score each patient received after the waiting period and immediately before the intervention, and ODI 3 is representative of the ODI score given after NeuroHAB, post-intervention (see Figure 3). No patient demonstrated competency in NeuroHAB-defined movement proficiency at the commencement of the intervention.

### 2.3. Exclusion Criteria

Patients were typical and representative of a specialist adult spinal neurosurgery practice in metropolitan Brisbane, Queensland, Australia. The age range was broad, ranging between 25 and 89 years old, and patients were tertiary referrals. Workers’ compensation back injury claims were not excluded and comprised 20 patients. The exclusion criterion from the NeuroHAB intervention was the presence of a deemed clinically assessable “movement-obstructing barrier” by the referring neurosurgeon. In the presence of a “movement-obstructing barrier”, movement therapy cannot be delivered.

Movement-obstructing barriers clinically preventing participation in NeuroHAB comprised the following:

1. Clinically and radiologically concordant intolerable symptomatic neural compression.
2. Motion segment structural or functional instability contributing to intolerable symptomatic mechanical low back pain and/or neural compression.

### 2.4. Oswestry Disability Index

Data was gathered using the Oswestry Disability Index (ODI) (see Table 1). The ODI is one of the most established and used measurement questionnaires for disability and pain in hospitals worldwide [39]. The comprehensive self-administered questionnaire has ten sections covering the severity of disability and its impact on daily life and activities. Referred to within the industry as the “gold standard” for measuring the quality of life [40], pain, and disability, the ODI system has a high test–retest reliability rate [41]

### 2.5. Statistical Analysis

The average change in ODI of the control-comparison group receiving usual care before commencing NeuroHAB was compared with the average change in ODI after the intervention. A single-factor one-way ANOVA (analysis of variance) was conducted using three data sets. Each data set represented the patient’s ODI score (pain and disability). A post-hoc analysis was conducted via multiple *t*-tests to establish pairwise comparisons between each data set. ODI 1 and ODI 2 were compared, acting as a waitlist comparison-control-type data set. ODI 1 and ODI 2 were then compared with post-intervention data, which was ODI 3. A Bonferroni adjustment was applied to the *p*-value initially set at 0.05. The Bonferroni adjustment involves dividing the current *p*-value by how many pairwise comparisons are calculated—this is designed to reduce the chance of causing a type 1 error and avoid a false positive. Additionally, basic descriptive statistics were calculated, among other valuable calculations, such as percentage comparisons between data sets and certain specific data.

## 3. Results

**Figure 4:**
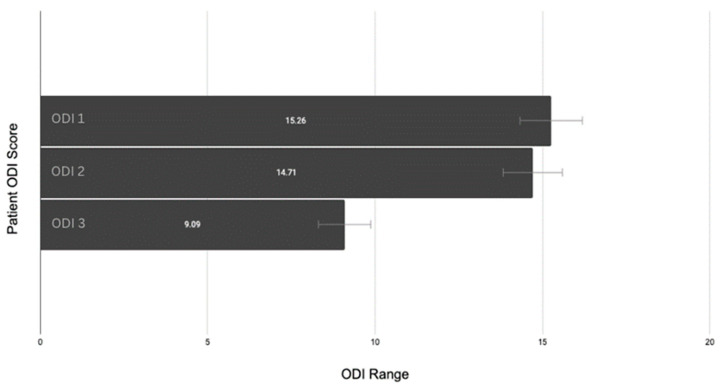
Patient ODI changes with confidence interval error bars.

Descriptive statistical calculations were conducted for an overall view of the data. The average ODI 1 score (consultation) was 15.26 ± 6.1% (CI: 14.3–16.2), ODI 2 (waitlist/pre-intervention) was 14.71 ± 6.0% (CI: 13.82–15.59), and ODI 3 (post-intervention) was 9.09 ± 8.6% (CI: 8.305–9.875) (see Figure 4).

A single-factor ANOVA of all three ODI data sets was conducted and indicated a significant difference between the data. The F value was 59.0, exceeding the F critical value of 3.0, with a *p*-value < 0.001. A post-hoc *t*-test pairwise comparison was conducted to determine any specific information of importance. The ANOVA indicated a significant difference, but to know which data set was significant, the three datasets had to be compared separately, in pairs. This allowed for the retrieval of helpful information, providing three *p*-values, one for each comparison. The *t*-tests were between ODI 1 and ODI 2, ODI 2 and ODI 3, and ODI 1 and ODI 3. The data between ODI 1 and ODI 2 were used as a control-like waitlist comparison, which was calculated to contrast the results between participants who did NOT experience the NeuroHAB program and from ODI 2 to ODI 3 for participants who then DID go through NeuroHAB.

Comparing the mean values from the first consultation (ODI 1) to those after the waiting list period and before the NeuroHAB program (ODI 2), no significant changes in ODI scores were present (mean difference: 0.55, *p*-value > 0.05). In contrast, the differences in mean values between ODI 1 (consultation) and ODI 3 (post-intervention) (mean difference: 6.17), and ODI 2 (post-waiting list, pre-intervention) and ODI 3 (post-intervention) (mean difference: 5.62) were significant (*p* < 0.001) (see Table 2).

A Bonferroni adjustment was also conducted to ensure accuracy and avoid type 1 errors. For every *t*-test calculated, the *p*-value of 0.05 must be divided. Three pairwise comparisons resulted in our starting *p*-value of 0.05 being divided 3×, resulting in a new *p*-value of 0.016666667. Pre-intervention ODI scores, compared to post-intervention scores, were both significant (see Table 3).

A reduction percentage was calculated between the ODI data and categorized yearly to further illustrate the significant changes in mean values.

**Figure 5:**
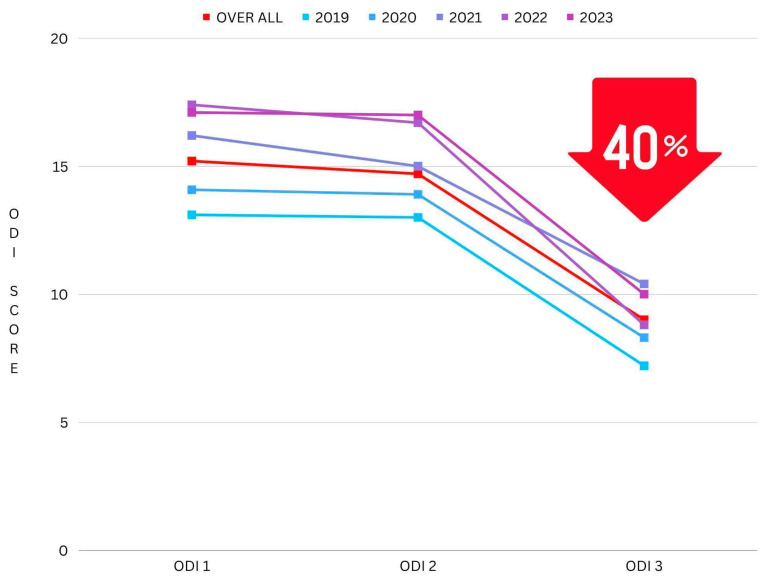
Reduction in ODI mean (grouped by year).

**Figure 6:**
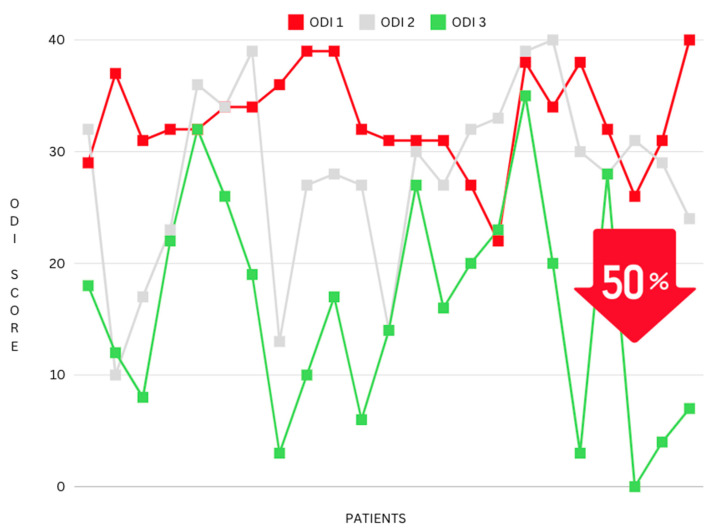
Reduction in ODI mean (grouped by the ODI category “Crippled”).

A 40.41% reduction can be seen between ODI 1 (the ODI score taken at the initial consultation) and ODI 3 (post-intervention) (mean difference: 5.62, *p* ≤ 0.001) (see Figure 5). Furthermore, a percentage reduction calculation was conducted on the most extreme cohort in this study, the “Crippled” ODI category, which ranges from 31–40 and is often explained as pain impinging on all aspects of the participant’s life. This cohort had even greater results, with a 50% reduction in pain and disability following the NeuroHAB program (mean difference 16.15, *p* ≤ 0.001) (see Figure 6).

Furthermore, standout participant improvements were also calculated as a percentage reduction, with the most notable ODI reductions showing a decrease of 74%, 82%, 91%, 92%, and 100% between ODI 1 (consultation) and ODI 3 (post-intervention).

### Waitlist Comparison Group (ODI 1–2) vs. NeuroHAB Group (ODI 2–3)

**Figure 7:**
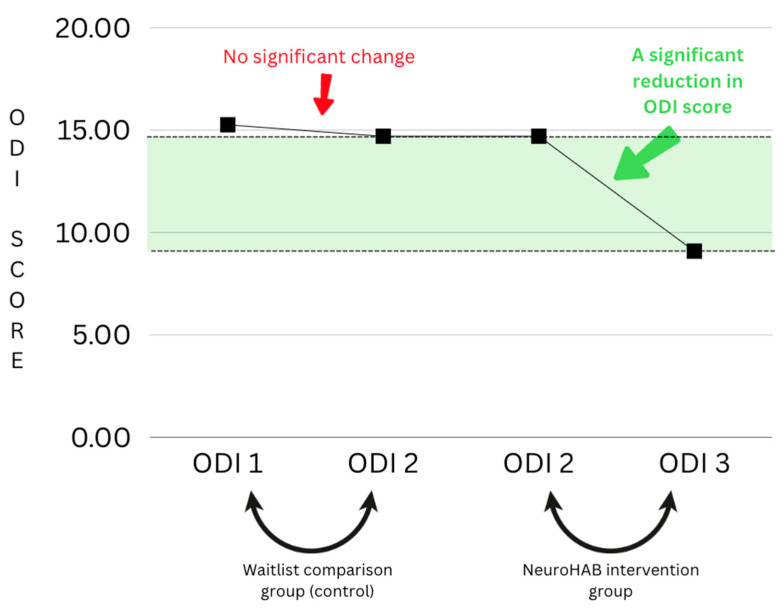
Waitlist comparison group graph.

This study was designed to utilize patients’ ODI scores from their first meeting (consultation) and contrast those with their ODI scores from after the waiting period and just prior to the commencement of the program. Each patient was required to wait an undisclosed period of time prior to starting the program; these ODI numbers serve as a control-like comparison group, as we can compare and contrast the ODI numbers of patients waiting (receiving no treatment) vs. those after the NeuroHAB program (the patient has finished treatment). For the purpose of this study, it is called the waitlist comparison group. Below, you can see there is no significant change from ODI 1 to ODI 2 (scores taken from the consultation vs. after the waiting period, before NeuroHAB). This was when the patient was on the waiting list. ODI 2 and ODI 3, however, display significant results, including a clear reduction in the ODI score, which represents a reduction in pain and disability. This change can be contrasted with the waitlist comparison group, which had no significant change at all. This is a clear indicator of the results retrieved from this research (see Figure 7).

Approximately 50% of patients referred for NeuroHAB declined the treatment recommendation for wide-ranging but uncategorized reasons. A primary theme for failure to participate was the inconvenience of time constraints associated with enrolling in a structured course of therapy and, secondly, being reassured that surgery was not indicated for their back pain condition. Non-compliance by these patients, who could be considered to “not be suffering enough yet”, likely further biased our study cohort to more severe pain and disability, further biasing poorer expected outcomes from the intervention.

## 4. Discussion

The purpose of this study is to document the improvement in pain and disability of 290 patients who underwent NeuroHAB Functional Movement Therapy between 2019 and 2023. In doing so, we have observed a significant change in disability and pain in patients undergoing movement therapy via NeuroHAB. Our examination of peer-reviewed literature has not identified any research defining movement dysfunction with the movement points of performance specified by NeuroHAB. In addition, no comparable studies describe an intervention with the distinctive intention of converting these coordinated and defined spinopelvic movement patterns into default movement patterns in patients suffering from chronic low back pain. This effectively treats the condition of movement dysfunction, which fundamentally represents the biomechanical disease that causes the massive majority of back pain symptoms currently unhelpfully labeled as non-specific low back pain, which by definition is a symptom, not a disease.

Frost et al. (2015) reported a difference in outcomes when examining firefighters over 12 weeks who were assigned movement-guided (MOV) fitness vs. conventional fitness (FIT). The MOV group was encouraged to maintain spinal control, a neutral spine, and neutral knee alignment when squatting, focusing on control and quality of movement. Compared to the FIT group and control group, the MOV group exhibited significant improvements in all areas of training and was the only group to improve spinal and frontal plane knee motion control. The authors concluded: “For occupational athletes such as firefighters, soldiers, and police officers, this implies that exercise programs designed with a movement-oriented approach to periodization could have a direct impact on their safety and effectiveness” [42]. In 2007, Kiesel et al., when investigating the Functional Movement Screen (FMS), found a correlation between FMS scores and injury in professional football players, stating, “A score of 14 or less on the FMS was positive to predict serious injury” [43]. Additionally, Williams et al. (2024) saw an improvement in movement potentiation scale and pain after 5 days of consecutive movement therapy, which focused on movement quality, balance, coordination, and optimal biomechanics [16]. Furthermore, Ikeda and McGill (2012) were able to provide low back pain relief through the modulation of motion, postures, and loads—stating that patients increased stability, increased mediolateral shear, and decreased spinal flexion [44]. Johnson and Kim (2023) described the prevalence of defined subclinical movement dysfunction of the lumbopelvic spine for simple bending tasks, supporting the fundamental concept of accumulative micro-injury over time progressively eroding stability and capacity, eventually emerging as persistent back pain symptoms when lifestyle functional demands exceed the individual’s functional capacity [38].

Maher et al. (2017) describe non-specific low back pain as both epidemic and having no patho-anatomical cause [45]. We question this logic on the pretense that, for any condition to become epidemic, it must, by definition, have a ubiquitous and simple root cause. A basic tenet of clinical medicine is to alleviate symptoms in conjunction with timely cause elimination. The likelihood of implementing an effective cure is low without understanding the disease process. This applies to chronic back pain, with a doubling prevalence between 1990 and 2015 due to symptom-focused interventions [1,2,3].

Given that NeuroHAB is a functional intervention characterized by skill acquisition, the maintenance of skilled movement proficiency is expected to continue to promote a further improvement in pain, disability, and musculoskeletal health beyond the movement therapy program, which is limited to 8 weeks. In the industrialized world, it remains highly plausible that the human musculoskeletal system performs simple modern tasks of daily living with ubiquitously poor movement proficiency. Fundamentally, the human musculoskeletal system, comprised of muscles, joints, ligaments, tendons, and bones, serves the primary function of movement [46,47]. A failure of the musculoskeletal system, including the lumbar spine, represents a primary failure of movement and, hence, the disease of movement dysfunction. Reinforcing this concept are the observed lower rates of low back pain (as well as hip and knee arthritis) and disability in non-industrialized cultures where toiling is higher [9,10,11,48]. At first, this may appear paradoxical, but when one recognizes that movement dysfunction is likely lower in these cultures, the irony is clear, exposing that movement proficiency protects against musculoskeletal pain [8]. Movement dysfunction is consistent with kinematically driving pathological biomechanical stress into a spinal motion segment, which drives biological inflammation, activating the cascade of nociception and accumulating structural changes in the musculoskeletal system or spine, referred to as spondylitis. This is distinct from the spinal movement void of movement dysfunction, which may have structural changes, which are correctly referred to as spondylosis or normal degeneration but which remain pain free due to the absence of activated structural adaptive nociception, inflammation, and secondarily maladaptive central pain pathways [42]. Holistic patient care requires the provision of specific and distinctive movement therapy for the disease of movement dysfunction, which causes the evolution of low back pain symptoms. The evolution is predictably from acute pain, then to episodic relapsing pain, and into chronicity and follows a cumulative dose–response relationship only tempered by symptom-based interventions, natural healing, and the integration of functional demand-reducing lifestyle restrictions, all of which fail to eradicate the underlying disease process.

### Current Back Pain Rehabilitation Guidelines

Currently, no guidelines describe intersegmental lumbar stability as the primary focus to reverse or prevent the epidemic condition of LBP [7,8]. Addressing and preventing the disease of movement dysfunction directly maintains lumbar spine stability and simplifies the myriad secondary spine pain-related syndromes and diagnoses such as flexion or extension intolerance, facet joint syndrome, sacroiliac dysfunction, discogenic pain, spinal stenosis, and disc prolapse, as well as mental health compromise and the layering of secondary central sensitization maladaptive pain, all of which, over time, add complexity to the simple inciting movement dysfunction root cause of back pain symptoms. The inclusion of functional movement proficiency and stability in future guidelines is a critical and necessary step towards meaningful improvement in epidemic levels of back pain-related clinical and economic morbidity.

## 5. Conclusions

Movement theory and the study of movement dysfunction present a paradigm shift in treatment centered on unified and consistent rehabilitation that defines proficient movement for the lumbopelvic spine. Movement theory and the clinically applicable model used in this study provide a window of opportunity for further research into functional movement therapy as the primary therapeutic methodology for eliminating the plausible disease of “movement dysfunction” that causes low back pain symptoms. The positive results of this first large series of 290 patients receiving NeuroHAB-defined movement therapy strongly support long-term and meaningful progress in the currently epidemic levels of chronic low back pain and its management.

### Limitations

This study has limitations due to the short follow-up period and the use of an internal waiting list comparison (control) group. A randomized control trial would offer an even greater level of evidence supporting a direct cause-and-effect relationship between our intervention and favorable outcome observations. The unique opportunity to document data from 290 patients over many years adds value to the body of research. This type of study, while not perfect by design, provides a unique and beneficial look at the benefits of defined movement therapy and opens up a promising avenue for future research on the subject.

## Figures and Tables

**Table 1:** Oswestry Disability Index (ODI).

Oswestry Disability Index (ODI)		
Interpretation	ODI Score	Percentage
Minimal disability	0–10	0–20%
Moderate disability	10–20	21–40%
Severe disability	21–30	41–60%
Crippled	31–40	61–80%
Bed-bound	41–50	81–100%

**Table 2:** ODI mean, SD, and % change between phases.

	ODI 1 Consultation (*n* = 290)	ΔI%	ODI 2 Waitlist/Pre-Intervention (*n* = 290)	ODI 3 Post-Intervention (*n* = 290)	ΔII%	ΔIII%
ODI	15.26 ± 8.12	−3.60%	14.71 ± 7.69	9.09 ± 6.82	−38.20%	−40.43%

Note. Values are expressed as the mean ± standard deviation; *n* = number of subjects; ΔI% = difference between consultation and waitlist/pre-intervention expressed in percentage; ΔII% = difference between waitlist/pre-intervention and post-intervention expressed in percentage; ΔIII% = difference between consultation and post-intervention expressed in percentage.

**Table 3:** Bonferroni adjustment.

Bonferroni Adjustment	
Current alpha	0.05
Bonferroni calculation	0.05/3
New alpha	0.01
ODI 1 vs. ODI 2	Not significant (*p* > 0.05)
ODI 2 vs. ODI 3	Significant (*p* < 0.001)
ODI 1 vs. ODI 3	Significant (*p* < 0.001)

## Data Availability

The original contributions presented in the study are included in the article, and further inquiries can be directed to the corresponding author.

## References

[1] World Health Organization Musculoskeletal Health. https://www.who.int/news-room/fact-sheets/detail/musculoskeletal-conditions.

[2] Bardin L.D., King P., Maher C.G. (2017). Diagnostic triage for low back pain: A practical approach for primary care. Med. J. Aust..

[3] Cieza A., Causey K., Kamenov K., Hanson S.W., Chatterji S., Vos T. (2021). Global estimates of the need for rehabilitation based on the Global Burden of Disease Study 2019: A systematic analysis for the Global Burden of Disease Study 2019. Lancet.

[4] Wu A., March L., Zheng X., Huang J., Wang X., Zhao J., Blyth F.M., Smith E., Buchbinder R., Hoy D. (2020). Global low back pain prevalence and years lived with disability from 1990 to 2017: Estimates from the Global Burden of Disease Study 2017. Ann. Transl. Med..

[5] Ferreira M.L., de Luca K., Haile L.M., Steinmetz J.D., Culbreth G.T., Cross M., Kopec J.A., Ferreira P.H., Blyth F.M., Buchbinder R. (2023). Global, regional, and national burden of low back pain, 1990–2020, its attributable risk factors, and projections to 2050: A systematic analysis of the Global Burden of Disease Study 2021. Lancet Rheumatol..

[6] Haas R., Gorelik A., Busija L., O’Connor D., Pearce C., Mazza D., Buchbinder R. (2023). Prevalence and characteristics of musculoskeletal complaints in primary care: An analysis from the population level and analysis reporting (POLAR) database. BMC Primary Care.

[7] O’Connell N.E., Cook C.E., Wand B.M., Ward S.P. (2016). Clinical guidelines for low back pain: A critical review of consensus and inconsistencies across three major guidelines. Best Pract. Res. Clin. Rheumatol..

[8] (2022). Low Back Pain Clinical Care Standard. https://www.safetyandquality.gov.au/sites/default/files/2022-08/low_back_pain_clinical_care_standard.pdf.

[9] van Dieën J.H., Reeves N.P., Kawchuk G., van Dillen L.R., Hodges P.W. (2019). Motor control changes in low back pain: Divergence in presentations and mechanisms. J. Orthop. Sports Phys. Ther..

[10] Stieglitz J., Buoro Y., Beheim B., Trumble B.C., Kaplan H., Gurven M. (2023). Labour’s pain: Strenuous subsistence work, mechanical wear-and-tear and musculoskeletal pain in a non-industrialized population. Proc. R. Soc. B Biol. Sci..

[11] Madan I., Reading I., Palmer K.T., Coggon D. (2008). Cultural differences in musculoskeletal symptoms and disability. Int. J. Epidemiol..

[12] Panjabi M.M. (1992). The stabilizing system of the spine. Part I. Function, dysfunction, adaptation, and enhancement. J. Spinal Disord..

[13] Panjabi M.M. (1992). The stabilizing system of the spine. Part II. Neutral zone and instability hypothesis. J. Spinal Disord..

[14] Haff G. (2010). Quantifying Workloads in Resistance Training: A Brief Review [Review of Quantifying Workloads in Resistance Training: A Brief Review]. Strength Cond. J..

[15] Johnson D.B., Feng L., Johnson C. (2023). Retrospective review of the efficacy for sublingual ketamine in the treatment of chronic low back pain defined by a cause and central functional pain symptom focused clinical model. Disabil. Rehabil..

[16] Williams B., Horschig A., Lock A., Johnson D. (2024). The movement potentiation scale and movement therapy—A study of three cases. J. Musculoskelet. Surg. Res..

[17] McGill S.M. (2001). Low Back Stability: From formal description to issues for performance and rehabilitation. Exerc. Sport Sci. Rev..

[18] Cook G., Burton L., Hoogenboom B.J., Voight M. (2014). Functional movement screening: The use of fundamental movements as an assessment of function—Part 1. Int. J. Sports Phys. Ther..

[19] Westcott W.L. (2012). Resistance training is medicine: Effects of strength training on health. Curr. Sports Med. Rep..

[20] Hessam M., Fathalipour K., Behdarvandan A., Goharpey S. (2023). The effect of mcgill core stability training on movement patterns, shooting accuracy, and throwing performance in male basketball players: A randomized controlled Trial. J. Sport Rehabil..

[21] Centre of Research Expertise for the Prevention of Musculoskeletal Disorders (CRE-MSD) There Is No Such Thing as “Non-Specific Back Pain”. https://uwaterloo.ca/centre-of-research-expertise-for-the-prevention-of-musculoskeletal-disorders/resources/position-papers/there-no-such-thing-non-specific-back-pain.

[22] Kahere M., Ngcamphalala C., Östensson E., Ginindza T. (2022). The economic burden of low back pain in KwaZulu-Natal, South Africa: A prevalence-based cost-of-illness analysis from the healthcare provider’s perspective. PLoS ONE.

[23] Potvin J.R., Mcgill S.M., Norman R.W. (1991). Trunk muscle and lumbar ligament contributions to dynamic lifts with varying degrees of trunk flexion. Spine.

[24] Callaghan J.P., McGill S.M. (2001). Intervertebral disc herniation: Studies on a porcine model exposed to highly repetitive flexion/extension motion with compressive force. Clin. Biomech..

[25] Aasa U., Bengtsson V., Berglund L., Öhberg F. (2022). Variability of lumbar spinal alignment among power- and weightlifters during the deadlift and barbell back squat. Sports Biomech..

[26] Wilke H., Neef P., Caimi M., Hoogland T., Claes L.E. (1999). New in vivo measurements of pressures in the intervertebral disc in daily life. Spine.

[27] Williams B.S., Johnson D. (2023). Low back pain Oswestry disability index changes following 8-week movement proficiency exercise program—A retrospective cohort study. J. Med. Res. Innov..

[28] Dr. David Johnson, Brain and Spinal Neurosurgeon and Back Pain Rehabilitation Specialist. https://www.youtube.com/watch?v=gPrcZRSTJrE.

[29] Nitz A.J., Peck D. (1986). Comparison of muscle spindle concentrations in large and small human epaxial muscles acting in parallel combinations. Am. Surg..

[30] Stevens S., Agten A., Timmermans A., Vandenabeele F. (2020). Unilateral changes of the multifidus in persons with lumbar disc herniation: A systematic review and meta-analysis. Spine J..

[31] Freeman M.D., Woodham M.A., Woodham A.W. (2010). The role of the lumbar multifidus in chronic low back pain: A review. PMR.

[32] Naghdi N., Mohseni-Bandpei M.A., Taghipour M., Rahmani N. (2021). Lumbar multifidus muscle morphology changes in patient with different degrees of lumbar disc herniation: An ultrasonographic study. Medicina.

[33] Hides J.A., Richardson C.A., Jull G.A. (1996). Multifidus muscle recovery is not automatic after the resolution of acute, first-episode low back pain. Spine.

[34] Hides J., Stanton W., Mcmahon S., Sims K., Richardson C. (2008). Effect of stabilization training on multifidus muscle cross-sectional area among young elite cricketers with low back pain. J. Orthop. Sports Phys. Ther..

[35] Le Huec J.C., Saddiki R., Franke J., Rigal J., Aunoble S. (2011). Equilibrium of the human body and the gravity line: The basics. Eur. Spine J..

[36] Hartmann H., Wirth K., Klusemann M. (2013). Analysis of the load on the knee joint and vertebral column with changes in squatting depth and weight load. Sports Med..

[37] Gallagher S., Pollard J., Porter W.L. (2010). Electromyography of the thigh muscles during lifting tasks in kneeling and squatting postures. Ergonomics.

[38] Johnson D., Kelly M. (2023). Defining the disease of movement dysfunction related low back pain—An observational study and description of the necessary paradigm shift required to cure the root cause of globally prevalent low back pain symptoms. J. Orthop. Res. Ther..

[39] Mehra A., Baker D., Disney S., Pynsent P. (2008). Oswestry disability index scoring made easy. Ann. R. Coll. Surg. Engl..

[40] Yates M., Shastri-Hurst N. (2017). The oswestry disability index. Occup. Med..

[41] Irmak R., Baltaci G., Ergun N. (2016). Long term test-retest reliability of oswestry disability index in male office workers. Work.

[42] Frost D.M., Beach TA C., Callaghan J.P., McGill S.M. (2015). Exercise-based performance enhancement and injury prevention for firefighters. J. Strength Cond. Res..

[43] Kiesel K., Plisky P.J., Voight M.L. (2007). Can serious injury in professional football be predicted by a preseason functional movement screen?. N. Am. J. Sports Phys. Ther..

[44] Ikeda D.M., McGill S.M. (2012). Can altering motions, postures, and loads provide immediate low back pain relief. Spine.

[45] Maher C., Underwood M., Buchbinder R. (2017). Non-specific low back pain. Lancet.

[46] McCuller C., Jessu R., Callahan A.L. (2020). Physiology, Skeletal Muscle.

[47] Nachemson A.L. (1981). Disc pressure measurements. Spine.

[48] Louw Q.A., Morris L.D., Grimmer-Somers K. (2007). The Prevalence of low back pain in Africa: A systematic review. BMC Musculoskelet. Disord..

